# Ecohealth research in Southeast Asia: past, present and the way forward

**DOI:** 10.1186/2049-9957-4-5

**Published:** 2015-01-29

**Authors:** Hung Nguyen-Viet, Siobhan Doria, Dinh Xuan Tung, Hein Mallee, Bruce A Wilcox, Delia Grace

**Affiliations:** International Livestock Research Institute (ILRI), 17A Nguyen Khang Street, Trung Hoa Ward, Cau Giay District, Hanoi, Vietnam; Centre for Public Health and Ecosystem Research (CENPHER), Hanoi School of Public Health (HSPH), 138 Giang Vo Street, Hanoi, Vietnam; Swiss Tropical and Public Health Institute (Swiss TPH), Socinstrasse 57, Basel, CH-4002 Switzerland; University of Basel, Basel, 4001 Switzerland; National Institute and Animal Sciences, Hanoi, Vietnam; Research Institute for Humanity and Nature (RIHN), Kyoto, Japan; Integrative Education and Research Programme, Faculty of Public Health, Mahidol University, Bangkok, 10400 Thailand; Division of Infectious Diseases and Global Health, Cummings School of Veterinary Medicine, Tufts University, North Grafton, MA 01536 USA; International Livestock Research Institute (ILRI), Nairobi, Kenya

**Keywords:** Ecohealth, One health, Southeast Asia, Interdisciplinary, Transdisciplinary, Scientific partnership, Capacity building, Network, Ecohealth content

## Abstract

Ecohealth is a comprehensive approach to understanding health at its human, animal and environmental interface in a socio-ecological systems context. This approach was introduced widely in Southeast Asia (SEA) by the Canadian International Development Research Centre (IDRC) in the late 2000s. Aimed at addressing the problem of emerging infectious diseases (EIDs), numerous such projects and activities have been generated throughout the region. Ecohealth is increasingly converging with the One Health approach, as both movements emphasise a holistic understanding to health. We conducted a scoping review by considering all of the Ecohealth programmes, initiatives and projects that have been implemented in SEA since the introduction of the approach, and also gathered information from peer-reviewed literature. The objective of this paper is to review Ecohealth activities within SEA over the last 10 years to address the lessons learned, challenges faced and the way forward for Ecohealth in the region. Activities range from those focusing purely on capacity, projects focusing on research and projects covering both. Achievements to date include, for example, research contributing to the field of infectious diseases in relation to social ecological factors and associated urbanisation and agricultural intensification. Challenges remain at the project design and implementation level, in the available capacity and coordination to develop Ecohealth research teams in the countries, gauging teams’ assimilation of Ecohealth’s underlying tenets and their translation into sustainable disease prevention and control, as well as in the ability to scale up Ecohealth projects. We suggest that the way forward for Ecohealth should be from a regional perspective in terms of research, training and policy translation using Ecohealth in combination with the One Health approach.

**Electronic supplementary material:**

The online version of this article (doi:10.1186/2049-9957-4-5) contains supplementary material, which is available to authorized users.

## Multilingual abstracts

Please see Additional file 1 for translation of the abstract into the six official working languages of the United Nations.

## Review

Ecosystem approaches to human health, or the Ecohealth approach championed by the Canadian International Development Research Centre (IDRC) is action-based research premised on the notion that human health and development depends on healthy ecosystems. Ecohealth approaches stress that the currently poor state of many of the world’s ecosystems is hindering efforts to improve global health and economic and human development [1]. While the IDRC began developing the Ecohealth approach in Latin America and Africa in the 1990s, its introduction in Southeast Asia (SEA) was more recent, during the late 2000s. The introduction of Ecohealth in SEA was largely stimulated by the emergence of avian influenza, Severe Acute Respiratory Syndrome (SARS) and other persistent zoonotic diseases in the region as SEA is a hotspot for infectious diseases. However, it is noted that Ecohealth is not only concerned with infectious diseases but also used to deal with wider environmental issues including chemical contamination. The dynamic landscape of research and application of the approach provides a wider space to address the interlinkages of health and the environment. These are particularly acute in SEA where rapid agricultural intensification, rural–urban transitions and climate change are having profound effects on ecosystems and health.

A similar multi-disciplinary, multi-sectorial and systems-based approach known as One Health was also widely promoted in the SEA in the early 2000s in the wake of the SARS and highly pathogenic avian influenza (HPAI) epidemics. The One Health approach shares similar values to Ecohealth and the parallels in both approaches have recently led to an increasing convergence of the two. This has led to a broader discipline incorporating infectious and non-infectious diseases, epidemiological and ecological methods, and disease control and development [2]. In SEA, One Health has been introduced mainly by USAID EPT (Emerging Pandemic Threats) RESPOND programme, and partly by the World Health Organization (WHO), the Food and Agriculture Organization (FAO) and the World Organization for Animal Health (OIE). As One Health activities are much more recent and have not involved research or intervention projects, they more frequently focus on network building. This is the reason that we did not examine One Health in depth but focused solely on Ecohealth, although these two approaches are similar. The objective of this paper is to review Ecohealth activities in SEA over the last 10 years to address the lessons learned, challenges faced and the way forward for Ecohealth in the region.

## Methods

For the purposes of this scoping review, we considered all of the Ecohealth programmes, initiatives and projects stimulated by or associated with the IDRC in SEA over the last decade. We obtained from the IDRC a list of all programmes, projects and initiatives funded by or affiliated with the IDRC Ecohealth Programme, as well as associated programmes utilising the Ecohealth approach. The main projects considered are shown in Table 1.

**Table 1:** **Ecohealth projects, countries involved and types of projects**

***Project name***	***Objective(s)***	***Countries involved***	***Project type***	***Field***	***Donor***
Asia Partnership on Emerging Infectious Diseases Research (APEIR)	Communication and knowledge sharing to reduce the threat of EIDs using a ’trust-based’ bottom-up approach. Focusing on avian influenza and expanded to other EIDs.	Thailand, Vietnam, Indonesia, China, Laos, Malaysia	Research	EIDs	IDRC
Ecohealth Emerging Infectious Diseases Research Initiative (EcoEID)	Understand the relationship between EIDs and agricultural, land utilisation and ecosystem management practices.	Thailand, Vietnam, Indonesia, China, Laos, Philippines	Research	EIDs	IDRC/DFATD/AUSAID
Ecosystem Approaches to the Better Management of Zoonotic Emerging Infectious Diseases in the Southeast Asia Region (EcoZD)	Increase the knowledge, skills, and capacity of research and infectious disease control personnel in SEA to understand the risks and impacts of Zoonotic Emerging Infectious Diseases (ZEIDs).	Vietnam, Laos, Cambodia, Indonesia, China, Thailand	Research–Capacity building	EIDs	IDRC
Eco-Bio-Social dengue control programmes	Combine the social and ecological dimensions of the emergence of dengue fever.	Thailand, Vietnam, Indonesia, China, Laos, Philippines	Research – Capacity building	Dengue	IDRC/WHO
Lawa Model: Integrated Opisthorchiasis Control in Northeast Thailand	Strategies for controlling the liver fluke infection using the Ecohealth/One Health approach.	Thailand	Operational Research	*O. viverrini*	IDRC
The Research Institute for Humanity and Nature (RIHN) project	Liver fluke infection in the lowland area of the Savannakhet Province in relation to the development of wet rice field and irrigation systems.	Lao PDR, Vietnam, Bangladesh, Yunnan China	Research	EIDs	IDRC
Building Ecohealth Capacity in Asia (BECA)	Building capacity in Ecohealth at different individual and institution levels.	Thailand, Laos, Cambodia, Vietnam, China	Capacity building	EIDs	RIHN
The Field Building Leadership Initiative in Southeast Asia (FBLI)	Research focus is on solving human health problems associated with agricultural intensification in SEA.	Thailand, Indonesia, Vietnam, China	Research – Capacity building	Agriculture and Health	IDRC
	Strengthen the capacity for Ecohealth research. Facilitate networks and knowledge sharing to mainstream Ecohealth and engage policy makers.				
Integrated assessment of environmental sanitation and health (NCCR North–South)	Develop a conceptual framework for improving health and environmental sanitation using an approach combining health, ecological and socio-economic assessments.	Vietnam, Thailand	Operational research	Agriculture and Health	SDC
Land Use Change and Human Health in the Eastern Himalayas: An Adaptive Ecosystem Approach	Reduce the vulnerability of mountain people to human health issues caused by land use change.	Nepal, Yunnan Province, Tibetan Autonomous Region of China		Agriculture and Health	IDRC

In addition, we conducted a literature search of peer-reviewed papers in electronic databases for the period up to June 2014. The purpose of the search and literature review was to assemble published articles and reports associated with these projects, as well as to identify any that were not affiliated with the IDRC or its partner agencies. The three main databases used in the search procedure were PubMed, ScienceDirect and ISI Web of Science. We employed the keywords: ’Ecohealth’, ’Ecosystem approach to health’, ’Southeast Asia’ and the specific names of Southeast Asian countries (e.g. Vietnam, Thailand, Indonesia etc.). These keywords were entered into the ’Title’, ’Abstract’ and ’Keywords’ fields in the databases. Through this search, we obtained a total of 70 results, which were screened for relevancy, resulting in a total of 21 papers that we analysed for this paper. In addition to the peer-reviewed literature, we also explored the grey literature on Ecohealth that was related to the Ecohealth projects mentioned above and found five reports that were not related to the IDRC projects. This included a review funded by the Australian Agency for International Development (AusAID), the National Centre of Competence in Research (NCCR) North–South programme and the US National Science Foundation (NSF). We also spoke to Ecohealth experts from Thailand, Indonesia, Philippines, Japan, Australia and Canada.

## History of ecohealth in the region

Ecohealth evolved in the mid-1990s as a paradigm conceived by IDRC scientists to better understand the linkages between nature, society and health [3]. Despite being relatively new and introduced in SEA in the mid-2000s, a comparatively expansive portfolio of activities has been stimulated. The first activity began in SEA in 2005 initiated by the IDRC, as a response to the outbreaks of SARS and avian influenza, with the establishment of the Asia Partnership on Emerging Infectious Diseases Research (APEIR) that is, as of 2014, active in five countries. The same year, the WHO Special Programme for Research and Training in Tropical Diseases (WHO-TDR) and the IDRC released a request for applications for the Eco-Bio-Social Dengue Initiative. This was launched in 2006 with a proposal development meeting held in Bangkok in early May 2006. In April 2007, in cooperation with the WHO-TDR, a workshop attended by research teams from six countries was organised and led by Pattamaporn Kittayapong and Bruce Wilcox at Mahidol University, Bangkok. The workshop represented a comprehensive introduction to the Ecohealth approach, including transdisciplinary ecological principles, the ecosystem concept and landscape mapping protocols relevant to dengue vector ecology.

Development of the workshop and training manual [4] was associated with a graduate education and research training programme (funded by the US NSF). This included the establishment of the first university course on Ecohealth and emerging infectious diseases (EIDs) entitled, ’Systems Ecology and Emerging Infectious Diseases’ in the Faculty of Sciences, Mahidol University, which is being developed and will be taught by Kittayapong and Wilcox.

The 2005 symposium, ’EID and Social Ecological Systems in Asia’, funded by the US National Institutes of Health’s (NIH’s) Roadmap to the Future Program, was an important stimulus for these and other activities, organised by the founding editor-in-chief and managing editor of the journal *EcoHealth*, Wilcox and Margot Parkes, respectively. This symposium included the participation of IDRC-affiliated scientists, as well as many of the academic leaders in Ecohealth at the time. The symposium’s outputs included a number of Ecohealth research syntheses and associated frameworks linking theory and practice [5–7], some strongly influencing activities in SEA. Interestingly, *EcoHealth’s* parent organisation, the International Association for Ecology and Health, also had a strong Asian link as its three founding officers, Wilcox, Parkes and Pierre Horwitz, were all based in the Asia-Oceania region at the time.

Subsequent research in Thailand focusing on mosquito-borne diseases [8, 9] and in Vietnam on avian influenza (H5N1) [10, 11] (the latter supported separately by the NSF’s Coupled Human Natural Systems Program) are framed explicitly on the basis of the EIDs research ’blue print’ that emerged from this symposium [7]. Fundamentally, the Wilcox-Gubler-Colwell model represents a social ecological systems framework for investigating the roles and interaction of urbanisation, agricultural intensification and habitat degradation, as well as their associated drivers and influences, in infectious disease re-emergence or emergence.

The above events that were scholarly in nature helped to both catalyse and lay the foundation for an expanding cluster of evolving academic research and graduate training activities in SEA. Distinct from these activities, although with some overlap of personnel and institutions, are projects principally championed by the IDRC, representing a combination of research, intervention, policy and capacity building. It is these distinctly ’ecosystem approach to health’ projects focusing on ’real life’ problems (in contrast to academic research questions) carried out largely outside university research settings that are the subject of this survey. Table 1 shows a summary of these Ecohealth projects with the main objectives as were stated in the project documents, as well as the type of projects they are.

## Ecohealth and emerging infectious research and policy

Southeast Asia is considered to be a hotspot for EIDs, specifically zoonotic and other vector-borne diseases [12, 13]. Rapid human population growth along with global and regional environmental changes are thought to be the main drivers, along with influencing factors including increasing human migration and global transport of people and goods, urbanisation, agricultural intensification and possibly climate change, are contributing to the re-emergence and newly emerging infectious diseases [13–16]. During the last decade, notable viruses, particularly SARS, avian influenza A H5N1, pandemic influenza A H1N1 and dengue fever, have attracted international attention and have had severe health and economic impacts in SEA [12, 13]. Projects throughout the region have worked to integrate an Ecohealth approach to the increasing (re)-emergence of infectious diseases to engage with their ecological and social factors.

In 2009, AusAID and the IDRC conducted a baseline assessment to evaluate past, current and emerging EID-related research for the Asia Pacific region [16]. It focused on country surveys in nine selected countries: Australia, Cambodia, Indonesia, Laos, Malaysia, Republic of Palau, Philippines, Thailand and Vietnam, to determine the extent of cross-sectional research examining Ecohealth and EIDs. The research team used a multi-stakeholder and multidisciplinary approach to collect, process, analyse and synthesise relevant data. The study found that there was a wide range in the extent of EID-related research and that there were gaps in knowledge about animal and environmental factors relating to the emergence of infectious diseases. Some of the barriers to research on EIDs were: a lack surveillance capacity and human resources, the reliance on foreign researchers, unreliable funding, minimal career incentives, and limited access to libraries and scientific journals. However, despite these challenges, the study found several integrated pilot or demonstration projects being conducted in SEA [16].

The Asia Partnership on Emerging Infectious Diseases Research (APEIR) was established 2006 to promote regional collaboration in avian influenza research. In 2009, as a response to H1N1 (swine flu), the network expanded its scope to promote Ecohealth and One Health concepts for all EIDs. Using a ’trust-based’ bottom-up approach, the APEIR works to facilitate knowledge generation, management, translation and capacity building to reduce the threat of EIDs [17, 18]. The APEIR has completed five IDRC-funded regional research projects that have yielded a number of outputs in published books, peer-reviewed scientific journal articles, reports, etc. [19–21]. Table 2 shows a summary of the research methods and findings of the completed projects. The APEIR is currently in its second phase with two ongoing studies (2013–2016) focusing on reducing biosecurity threats from infectious diseases and on the proper use of antimicrobials in humans and animals to control antimicrobial resistance in SEA. Research findings from these two studies have not yet been produced.

**Table 2:** **The APEIR completed projects**

***APEIR sub-project***	***Research issues***	***Research method***	***Findings (where applicable)***
*Migratory birds & AI network*	What is the role of birds in spreading AI?	Documentary reviews; Wild birds capture and identification; and Cloacal/tracheal swabs, serology sampling GIS, satellite tracking.	It is not clear whether the wild birds are the source of poultry infection; major wild bird migration routes along the central Asia flyway overlap with areas that have experienced avian influenza outbreaks in poultry in Tibet.
	What are the AI viruses in wild birds?		
	What species of birds are infected?		
*Socio-economic impacts of AI*	How households’ livelihood and wellbeing are affected by AI and AI control measures.	Cross-country comparisons of household level data; Structured-interviews/ group discussions; and ST social, economic issues related to AI control.	The backyard poultry sector is resistant to shock but the small-scale commercial sector is vulnerable.
			Farmers considered the compensation rate for culling of poultry during the HPAI outbreak to be inadequate – discouraged farmers to apply control measures and not hide/sell their infected poultry.
*Backyard poultry systems & AI*	What are characteristics & dynamics of BP systems?	Mixed methods: farmer interviews, direct observation, focus group discussions (FGDs); Design and test models; and Cross-country comparisons.	Data on the characteristics and economics of smallholder and backyard producers provided valuable information for policy makers.
	What are the marketing networks?		Biosecurity is generally quite low in both smallholder and larger commercial farms.
	What are some effective/feasible ways to reduce AI risk?		
	How can these be tested in practice?		
*Policy analysis*	What are antiviral drug and poultry vaccination policies?	Documentary search; Stakeholder analysis; Semi-structured interviews; A series of meetings with key informants to explore issues in policy formulation and sequence of events surrounding them); and Cross-country comparisons.	Scientific evidence plays a role in related discussions, but national economic interest is important.
	What are the contextual factors influencing/the process and development of policies?		Technical information on use of vaccination of poultry for H5N1 HPAI was interpreted differently in Thailand as compared to Vietnam and Indonesia, resulting in different conclusions on its utility.
	What are differences and similarities in policy/context/process among the three countries?		
*Effectiveness of AI control measures*	What measures have been recommended & implemented?	Literature reviews to prioritise CMs FGDs and observation; Risk assessment & estimate effect of control measures(CMs) on risk; Province in-depth case studies; Cross-country comparisons; and Farms & districts as systems.	Control of highly pathogenic avian influenza was achieved, despite poorly implemented control measures.
	How have these been implemented?		Vaccination in Vietnam and China did not prevent all cases of infection, but played a role in reducing disease levels.
	What is impact on risk reduction?		
			Poultry vaccination has reduced the occurrence of outbreaks in Vietnam and China, but it may be masking virus presence.
			Reliance on mass vaccination is leading to neglect of other measures.
*Poultry Production Clusters (PPCs)*	What is impact of these PPCs on the socio-economic status of the producers?	Survey on impacts of PPCs (PRA case study, FGDs, interview, observation), pilot intervention in PPCs; Dissemination and advocacy; and Look at impact on all related aspects.	Poultry Production Clusters (PPCs) have developed industrial production of poultry, improved farms’ economic efficiency and controlled diseases.
	What changes in attitudes, behaviours and relations among various stakeholder groups induced by the development of PPCs?		Evidence of economies of the scope for PPCs in terms of access to feed at lower cost and more stable output price. Farms benefit from social changes in PPCs through better information sharing and cooperative activities in feed use, disease control and infrastructure development.
	What are Ecohealth pilot interventions to improve the livelihoods of small producers?		
			Poultry inside PPCs are less likely to get infected with diseases.
*Small-Scale Poultry Slaughter Houses*	What is the hygienic status in small-scale poultry slaughterhouses and their effects on ecological and health in the community?	Interviews, FGDs; Observation; Collecting the sample from slaughterhouse (SH)/laboratory test systems: SH, traders, retailed, people living around the SH.	High prevalence of *Salmonella* contamination of carcasses in the poor hygienic conditions of the small-scale poultry slaughter houses.
			Serotyping revealed the presence of *S. enteritidis* and *S. typhimurium*, which were potentially food poisoning microorganisms, and presumably contaminated from poultry flora due to slaughtering performance.
			Water source and waste appeared to be the most important factors correlating to the *Salmonella* contamination.

The Eco-Bio-Social Dengue (EBS) Initiative ran largely parallel with the first APEIR projects, though it ended in 2011 [22]. The initial aim of the EBS Initiative was to contribute to ’improved dengue prevention by better understanding its ecosystem-related, biological and social (’eco-bio-social’) determinants and to develop and evaluate community-centred ecosystem management interventions, embracing public intersectoral actions, to reduce dengue transmission below threshold levels for epidemic outbreaks’ [23]. The six country teams selected for participation were from India, Sri Lanka, Indonesia, Myanmar, Philippines and Thailand. The teams met together with the TDR project managers and one of more external advisors in workshop settings annually in different locations in Asia hosted by country teams. The TDR referred to these as community of practice (CoP) meetings, with the intent of facilitating the establishment of a regional community of researchers interested in applying the Ecohealth approach to dengue control.

The country projects, each focused on urban or peri-urban study areas with a history of high incidence of dengue, and followed a core protocol including conceptual framework for the eco-bio-social approach, along with specific sampling design provided by the WHO-TDR [23]. The approach combined standard methods from an epidemiology study design for household demographic and larval surveys with novel social science-based community participatory methods. It was interdisciplinary (did not employ a transdisciplinary approach), seeking to combine disciplinary methods that were complementary. The conceptual framework and study design was based largely on the WHO’s Integrated Vector Management (IVM) approach with added attention to socio-cultural aspects pertinent to community participation. It did not incorporate the ecosystem concept nor was it framed using a systems perspective [23].

The projects under the EBS Initiative were originally planned for two years: a one-year study and an intervention planning phase followed by an implementation phase in the second year. As prescribed by the WHO-TDR, each of the country teams undertook a situational analysis. This included environmental characterisation and mapping of their study areas, and assessing vector abundance in relation to season and breeding container types. This was complemented by data and its analysis of the social and cultural context, with the objective of understanding stakeholder community and gender implications for vector control. The intervention phase using tools appropriate to local contexts were selected and employed cooperatively with community participants. Measurable outcomes were achieved locally in both the reduction of mosquitoes and the community participants’ interest and understanding of effective vector control measures. Country project outcomes are summarised by Sommerfeld and Kroeger [22] with accompanying articles in the same issue. It is not clear from the report whether the control efforts put in place will be sustained.

The Ecohealth Emerging Infectious Diseases Research Initiative (EcoEID), funded by the Department of Foreign Affairs, Trade and Development Canada (DFATD), AusAID and the IDRC, was launched in 2010 to enhance the response capacity to the threats of EIDs. After a competitive call for research proposals, three multi-country and multi-disciplinary research proposals spanning seven countries in SEA were selected [24]. The first of these, ’Innovative Strategies for the Sustainable Control of Asian Schistosomiasis and Other Helminth Zoonoses’, focuses on diseases caused by parasitic worms. Its aim is to better understand disease risk, including the ecological and socio-economic determinants of transmission in local contexts. Cost-effective interventions to increase community awareness and enhance local response capacity will then be implemented on this basis. The second project, ’Application of an Eco-Bio-Social Approach to Emerging Infectious Diseases in Southeast Asian Global Outreach Hotspots’, aims to explore the dynamics between EIDs, tourism and development. The project has three objectives: (1) to change local health and development policies, (2) to create integrated disease surveillance systems in tourist destinations, and (3) to strengthen the capacity to develop integrated and multi-sectorial intervention approaches [25]. The final selected project, ’Poultry Production Clusters’, will examine the impacts of concentrated poultry production on farmers’ livelihoods, the environment and disease risks. The project aims to build greater government support for local poultry farmers, improve the livelihood of the farmers, strengthen national and regional policies on poultry production and enhance the understanding of how agriculture can contribute to improved health. These three projects are ongoing, with completion dates scheduled for the end of 2014.

The ’Ecosystem Approach to the Better Management of Zoonotic Emerging Infectious Diseases in the Southeast Asia Region (EcoZD)’ was a programme operated by the International Livestock Research Institute (ILRI) with funding from the IDRC from 2007 to 2013. It aimed to promote and facilitate sustainable management practices for priority and emerging zoonoses, and to strengthen networks of stakeholders to develop capacities and communication strategies [26]. Applying ’a learning by doing’ Ecohealth and One Health approach, the completed project produced multiple research outputs in six pilot countries. Table 3 provides a brief overview of the country specific projects and the Vietnam team also provided an excellent example of utilising various approaches to addressing interdisciplinary strengthening and differences in priorities. At the project level, behaviour changes in country teams were documented in three key areas: understanding and applying Ecohealth principles, communication of research findings as a part of stakeholder outreach and the knowledge translation process, and networking and policy engagement [27]. Although the program formally ended, related activities continue on a smaller scale, funded by the CGIAR Research Program on Agriculture for Nutrition and Health [28].

**Table 3:** **Overview of country-specific EcoZD projects**

	***Research focus***	***Research methods***	***Findings***
***Cambodia***	Risks of zoonotic diarrhoea in rural households	Household questionnaires and biological sampling.	Humans were rarely isolated from animals facilitating disease transmission.
***Indonesia***	Generating evidence on dog movement and behaviour	Surveys for dog demography, fecundity, movement and gathering socio-cultural data.	Disproved that dogs were spreading rabies across the island and that culling dogs would control rabies.
***Lao PDR***	Pig zoonosis	Questionnaires and biological sampling of serum for pigs and humans.	Zoonoses from pigs were common in rural areas Much of the disease burden was related to poor awareness.
***Thailand/Vietnam***	Microbiological contamination in poultry and water	Quantifying the microbiological contamination; Focus groups.	*Salmonella* spp. was an important hazard for small-scale chicken slaughterhouses.
***Vietnam***	Exposure to leptospirosis	Retrospective study; Questionnaire; and Biological sampling.	Exposure was common but infected pigs did not pose a significant risk to humans; rather both pigs and people had risk factors related to environmental sources of infection.
***China (Yunnan Province)***	Whether brucellosis was a zoonotic emerging disease in the Yunnan Province	Questionnaire; and Biological sampling of blood and mile from animals/humans.	Brucellosis remains uncommon.
			There is low awareness of the diseases.

In 2008, the Research Institute for Humanity and Nature (RIHN) launched Ecohealth projects in SEA to examine the relationship between endemic infectious diseases, ecosystem and societal transformation. In Lao PDR, the RIHN engaged multiple partners to conduct studies on liver fluke and malaria infection in the Savannakhet Province [29]. Small liver fluke infection was studied in the lowland area in relation to the development of wet rice fields and irrigation systems. Study results from this project show that deforestation has led to the expansion of wet rice fields and irrigation and an increase of population density, thus increasing the population at risk of *Opisthorchis viverrini* infection [29]. In the mountainous area, a trans-border study of the Laos-Vietnam border was conducted to examine malaria in relation to forest degradation. These results found a large difference in malaria incidence. This project established two Health and Demographic Surveillance Systems (HDSSs) and one mobile phone network in the province, both which continue to produce Ecohealth data [29].

The ’Lawa Model: Integrated Opisthorchiasis Control in Northeast Thailand’ is an ongoing effort initiated in 2008 by the Tropical Disease Research Laboratory, a unit affiliated with the Department of Pathology, Faculty of Medicine, Khon Kaen University (KKU-TDR), Thailand. The KKU-TDR’s focus has been village-based cholangiocarcinoma (CCA) and *O. viverrini* screening of volunteer community members, provision of antihelminth medication at no cost and education focused on discouraging consumption of uncooked fish. Sripa et al. (2015) provide information on the history of the control efforts in Thailand and the Lawa project [30].

The Lawa Model represents a significant elaboration of the Thai government’s top-down nationwide liver fluke campaigns conducted since the 1980s that failed to impact much of the Northeast. Concentrating on ten villages near Lawa Lake in the Khon Kaen Province, KKU-TDR’s programme has apparently resulted in significant reductions in prevalence, locally. The recognition of a general association of *O. viverrini* infection prevalence and CCA incidence with populations whose livelihoods are tied to reservoir-wetland ecosystem complexes represented an important research contribution [31].

Particularly in light of this, associating with EcoEID in 2011 and incorporating elements of the Ecohealth approach offered the opportunity to develop the Lawa Project as a model for opisthorchiasis control in the lower Mekong Basin. Significant challenges in developing an integrated control strategy include overcoming gaps in the scientific understanding of *O. viverrini* transmission and its contribution to disease. Details of the transmission dynamics of *Opisthorchis spp.*, particularly regarding ecology and social ecology, remain largely unknown. The same is true for the health risk of the *O. viverrini* infection that raw fish consumption actually poses in terms of CCA, as other dietary factors as well as genetic polymorphisms may be more important (see [32] and references cited therein). The KKU-TDR, among other research groups, has recently initiated research to address these gaps.

## Ecohealth and agricultural intensification, and environment and climate change

Southeast Asia is experiencing rapid agricultural and livestock intensification, which is having a profound impact on ecosystems and human health [13]. Intensive use of chemical fertilisers, pesticides and irrigation technology, in combination with high-yielding crop varieties is placing intense pressures on the environment and its natural resources [33, 34].

An early Ecohealth project that had great influence in SEA was the ’Land Use Change and Human Health in the Eastern Himalayas’, initiated in 2006. Supported by the IDRC, the International Centre for Integrated Mountain Development (ICIMOD) conducted action-based research to understand the links between land use change and human health in the eastern Himalayas. The overall objective of the project was to reduce the vulnerability of mountain people to human health issues caused by land use change. The research was conducted in three field study sites in Nepal, the Yunnan Province and the Tibetan Autonomous Region of China, each with a number of research objectives, questions and hypotheses. Specifically in the Yunnan Province, the focus of the project was on water improvement and pesticide management in the context of agricultural intensification. The multi-disciplinary team used several methods to collect data in four villages across three townships. The results of the study showed that commercial vegetable farming strains limit water resources and bring new challenges to the local management regimes. Intensified vegetable cultivation has led to inadequate water supplies from the river, creating tensions over water allocation. An additional stress on the water is the pollution caused by chemical fertilisers, pesticides and domestic waste. Many water sources lack basic structural protection, increasing the risk of contamination. The study concluded that water shortages would continue to worsen with the expansion of vegetable cultivation [35].

The National Centre of Competence in Research North–south (NCCR North–South) is a Swiss research programme that focused on global change and its impact on sustainable development. Launched as a 12-year program in 2001, the NCCR aimed to build research capacity and partnerships between nine institutions throughout Asia, Africa, Latin America and Switzerland. In SEA, the NCCR North–South research focused on the health impacts of environmental sanitation. An Ecohealth research programme was implemented from 2007 to 2012 in the Hanam Province, Vietnam to comprehensively assess the impact of combined human and animal sanitation [36]. Hanam, a peri-urban study site, offered a good setting to study a system combining human and animal sanitation, as in this area human excreta and animal manure are used together with wastewater in agriculture and aquaculture [37]. Most households (85%) engage in agricultural activities; they are predominantly smallholders and often raise 2–20 pigs on land that is simultaneously residential, agricultural, aquacultural and horticultural. The use of waste raised issues for environmental sanitation, agriculture, and health and well-being. Three components of the framework were implemented, namely environmental, health and socio-economic assessments, leading to the identification of critical control points with an important emphasis on participation of the community [38].

The Field Building Leadership Initiative in Southeast Asia (FBLI), a five-year (2012–2017) regional programme, is working to address ecosystem and health issues related to agricultural intensification [39]. Implemented by seven institutions and universities in Thailand, Indonesia, Vietnam and China, the initiative uses a diverse range of methods and activities, including the ’site-based research concept’ in Yuanmou (Kunming, China), Hanam (Vietnam), Chachoengsao (Thailand), and Pangalengan and West Java (Indonesia). Research is the backbone of the initiative and serves as a platform for future growth in the field. While each project site focuses on a research topic related to individual country priorities, the ultimate impact is seen through more sustainable agricultural practices and livelihoods. The long-term vision of the programme is to develop a well-established field of Ecohealth that is sustainable, institutionalised, and influential in driving environmental and health policy [40]. Although the FBLI is still at an early stage to harvest research findings, Table 4 provides a summary of the key findings from country-specific situational analysis, stakeholder workshops, Participatory Rural Appraisal (PRA) and preliminary surveys.

**Table 4:** **Summary of early findings from country-specific FBLI projects**

	***China***	***Indonesia***	***Thailand***	***Vietnam***
***Site***	Yuanmou Country, Yuanmou Province	Pangalengan, Bandung District, West Java Province	Chachoengsao Province	Hanam Province
***Entry points***	Controlling pesticide use; and Promoting better water management practices.	Dairy production; Connecting issues; and Finding interventions for small-scale farming.	Proposed best practices among communities associated with rubber plantations to reduce their risk of vector-borne diseases.	Livestock and human waste recycling for agriculture; and Impact on human and environmental health.
***Research methods***	In-depth interviews; Household questionnaire survey; Laboratory test of pesticide residues of vegetable and fruit samples; and Data analysis.	Literature search; Pre-survey questionnaire; In-depth interviews and FGDs; and Data analysis.	Situation analysis; Specific field site visits; and Preliminary survey and questionnaire.	Qualitative scoping/interviews; Participatory stakeholder workshop.
				Secondary data collection; Samples collected; Data was compiled and initial analysis.
***Findings (early results as of January 2014)***	Two types of farmers in Yuanmou: local farmers and farmers who work on farms and plantations as daily wage labourers.	Nearly all farmers in the groups owned their land, farm and cows.	Malaria, dengue and chikungunya are an issue in the province; 60% of malaria reported was found in labourers, possibly working in the rubber plantation.	The number of households with livestock decreased but the number of livestock heads (pigs) increased; Common method to manage animal waste is biogas; Community has concerns with pesticide use in cropping and manage package after use; and Communes do not have landfill or treatment sites.
	Results from pretesting reveal that around 10% of the samples tested positive for pesticides.	Main problems are low-quality concentrates, a lack of grass and other foodstuffs and poor management of small farms.		
		Poor productivity and quality of the milk mean farmers must accept very low prices for their milk.		
		Some farmers may dilute their milk before sending it to collection stations.		

## Network development, capacity building and training

The projects explored in this paper aim to build capacity of researchers and institutions in order to effectively promote and scale up the Ecohealth approach. Through numerous workshops and exchanges, the national research teams of the APEIR have been able to jointly design, plan and implement their projects and have been recognised for their role in regional collaboration [17]. The EcoZD project met its main objective of increasing the capacity of researchers and implementers to use an Ecohealth approach for better control of zoonoses, proven through outcome mapping (OM)^a^ and Ecohealth uptake assessment [26]. The project exceeded its goals by developing two Ecohealth Resource Centres (EHRCs) that introduced manuals, short courses and training for students and practitioners. Another EHRC has been developed at the Hanoi School of Public Health from a post-doctoral research that focused on interdisciplinary projects [41]. From the onset of the FBLI, the programme aimed for comprehensive capacity building. Activities included the development of an Ecohealth Trainer Manual, training of Ecohealth trainers, training a generation of future Ecohealth leaders (True Leaders Program) and introducing graduate degree training programmes at the MSc and PhD level in Ecohealth, One Health and Ecosystem Management [42].

Contributions from different Ecohealth projects in the region have stimulated Ecohealth teaching at universities in SEA and East Asia. Mainly these take the form of short courses or certificate training and integrating Ecohealth into existing curricula. Table 5 summarises Ecohealth courses taught in universities in the region. The courses have attracted hundreds of students each year, but as graduate and post-graduate courses are relatively new, there little information on the demand for ’Ecohealth’ or ’One Health’ graduates.

**Table 5:** **Ecohealth courses taught in universities in the region**

***Training***	***Host institution***	***Countries and partners involved***
Ecohealth degree trainings:	Mahidol University (pending)	Thailand, Vietnam, Indonesia, China, Laos, Malaysia
• MSc in One Health and Ecosystem Management	CMU (with UMN)	
• MSc in Global Health		
Ecohealth short courses:	HSPH, KMU, UI, Mahidol, UGM, CMU, ILRI, VWB/VSF	Thailand, Vietnam, Indonesia, China, Malaysia
• Concept of Ecohealth		
• Ecohealth and risk assessment		
• Ecohealth and food safety		
• Global Health True Leader		
• Emerging Zoonotic Diseases (EZDs)		
Integrated Ecohealth in existing modules:	HSPH, KMU, UI, Mahidol	Vietnam, Laos, Cambodia, Indonesia, China
• Environmental Health		
• Epidemiology		
• Food safety and nutrition		

CMU: Chiang Mai University, Thailand.

UGM: Gadjah Mada University, Indonesia.

UI: Universitas Indonesia, Indonesia.

KMU: Kunming Medical University.

HSPH: Hanoi School of Public Health.

ILRI: International Livestock Research Institute.

VSF: Veterinarians Without Borders, Canada.

Other fora and symposia in the region of SEA have contributed to the networking and development of new ideas for promoting Ecohealth. For example, several special sessions or side events were organised at the Prince Mahidol Award Conference (PMAC) in Bangkok in 2013 and 2014. Other Ecohealth symposia were organised in Hue, Vietnam and Beijing (China) in collaboration with the RIHN at the 4th Asia Pacific Conference on Public Health ’Climate Change and Population Health’. The first regional Ecohealth of Asia Pacific’s by Veterinary Public Health Centre for Asia Pacific (VPHCAP) conference was held in Chiang Mai in 2011 [43]. In October 2012, the fourth biennial conference of the International Association for Ecology and Health was held in Kunming, China. This meeting had large group discussions about the emerging field of Ecohealth and how best to create a sustainable environment for Ecohealth-type research. The diversity of conference attendees allowed for an in-depth analysis of the field and showed the need for connections between groups and enabling structures that support conversations and collaborations between groups [44].

Building Capacity for Ecohealth Research and Practice in Asia (BECA) is a research project that investigates the processes involved in building capacity for research and application of ecosystem approaches. Through workshops, training events and resource development, BECA aims to develop the capacity to reduce the risk of outbreaks of EIDs in Cambodia, China, Indonesia, Lao PDR, Thailand and Vietnam [45]. BECA also developed the capacity of partners to write policy briefs from research evidence for informed policy.

## Paradigm shift: from proactive support of donors to competitive process for funding

As mentioned above, although some Ecohealth projects in SEA have been financially supported by AusAID, the WHO and the Swiss Agency for Development and Cooperation (SDC), major Ecohealth projects on EIDs and ZEIDs have primarily been funded by the IDRC. The sustainability is questionable; for example, when Ecohealth does not exist as a programme at the IDRC, the question arises as to how Ecohealth research will be supported by other donors besides the IDRC. A pragmatic response by partners has been to brand themselves as both Ecohealth and One Health (e.g. the Chiang Mai resource centres), signalling to a broader range of donors.

The process of IDRC grant making is to guide, in a participatory fashion, the potential grant recipients (selected on the basis of pre-proposals) to develop an acceptable final proposal, upon which the project’s funds are released. For many of the other donors, the topic of projects is set by the funder, which leaves little room for partners to identify locally relevant topics that fit local needs. For example, the topic of EIDs is internationally important, however, it might not be among the top priorities of some countries and regions from the perspective of burden of diseases and social impacts. Interestingly, an assessment by the EcoZD project found that many local partners accepted the priorities of the international community (e.g. avian influenza), while simultaneously stated that other priorities (e.g. food-borne diseases) were of far greater importance to the communities they served.

More recently a shift has been observed towards a more competitive basis and grantees have more liberty to identify relevant research topics. The EcoEID programme had two rounds of calls for concept notes and full proposals, and the partners identified the research questions from their perspective, while staying within the topic of infectious diseases. In this case, the IDRC had a smaller role in proposal development. Three major EcoEID projects were selected [46]. Another initiative of Ecohealth, the FBLI project, was able to mobilise a regional consortium to develop a non-infectious focused research – training – intervention programme on agricultural intensification [40]. In this FBLI development, the IDRC also collaboratively partners through a proposal development process of different stages, but it is the partners of the consortium who mainly identified the main research and training topics. It was probably due to this proactive role of the consortium that the initiative did not address infectious diseases but rather animal waste management, dairy and food safety, pesticide use, and rubber plantation and health [42]. It is interesting to see this shift in funding modalities and the way researchers in Ecohealth become more proactive in proposing their ideas to donors. This can be seen as an indicator of the sustainability of Ecohealth ’in practice’ in the region, as Ecohealth is applied to issues locally identified by partners.

## Conclusion main challenges and the way forward for ecohealth in sea

Ecohealth and the closely related One Health, both of which can be characterised as integrative approaches to health, have proved to be highly attractive concepts that easily win approval from a wide range of stakeholders. Integrative health approaches are endorsed by the ’three sisters’ (the WHO, the FAO and the OIE) viewed as the three global standard setters for health, zoonoses and food-borne diseases. Important publications have gathered evidence that integrative health research is more effective and accessible than sectoral approaches, and a broad range of journals and conferences disseminate research on Ecohealth and One Health. Globally, there is a need to develop an understanding of the dependence of health on ecosystems, as well as a need to widen Ecohealth approaches to address the interconnected and up-stream drivers of health and well-being: environmental, social and economic factors [47].

These imperatives for more integrative health approaches notwithstanding, the great majority of medical education, clinical practice, ancillary services, development programmes and research continue to operate within disciplinary boundaries. Grace et al. (2011) identified ’barriers and bridges’ in this regard in relation to Ecohealth in SEA [48]. Among the barriers were: many of the Ecohealth successes have been ’boutique projects’ which did not demonstrate impact at scale; enthusiasm for Ecohealth has been highest among veterinarians, environmentalists and medical sociologists who are the most marginalised in the arena of human health; although the epidemiologic rational for Ecohealth is well demonstrated, economic and policy aspects have received less attention; cultural attitudes can promote hierarchies and gate-keeping of knowledge rather than more egalitarian and open approaches typified by Ecohealth; and concerns that Ecohealth is one of a large number of new paradigms that originate outside SEA.

In spite of these barriers, overall success of Ecohealth in SEA is demonstrated by the scope and scale of activities collectively encompassed by the projects, programmes and initiatives reviewed here. Ecohealth has been both widely accepted and gained a remarkable amount of exposure in SEA in a relatively short time. Still, limited human and financial resources, as well as a lack of capacity and coordination to develop Ecohealth research, are obstacles to scaling up the Ecohealth approach. Overall, achieving policy influence remains a constraint to the success of the Ecohealth approach [47].

The Ecohealth approach presents new challenges and opportunities for researchers, community-groups and policy makers. While Ecohealth is an innovative approach that is aligned with the latest thinking in global health and international development, the field faces several barriers to implementing sustainable change [3]. The question that remains is whether the interest and activity will continue without funding explicitly directed toward Ecohealth, One Health or similar approaches in the absence of a relevant level of funding during the past decade. The answer may lie in how well these concepts have been understood, integrated within or have been adapted to be complementary to previously existing missions, programmes or agendas of the above institutions and organisations.

However, much remains to be done before Ecohealth can be considered reasonably operational or institutionalised. The measurement of its progress in this regard, as well of outputs in terms increased human resource capacity in the form of knowledge and skills required to implement Ecohealth approaches based on Ecohealth tenets is beyond the scope of this paper. This is essential to generating evidence for the sustainable benefits of Ecohealth. The project descriptions provided here, which are based largely on information self-produced and thus inevitably subjective to some degree, can only provide glimpses of this. Clearly, projects varied in the degree to which their components, design or methods were consistent with a transdisciplinarity approach. Thus it has not been possible to objectively assess from our survey how well these ideas are understood conceptually, and especially whether this understanding is sufficiently comprehensive to serve as a solid foundation for continued development. More information on each project, along with specific evaluation criteria is required to truly gauge their impact, such as that provided by Wilcox et al. [49], who offer a list of seven components of a successful Ecohealth project.

In addition to such evidence, promoting Ecohealth approaches requires an understanding of how major shifts in health policy and practice occur, as well as how the ability to influence opinion and policy shifts. Many models exist for both understanding and influencing policy change and most of these involve, either implicitly or explicitly, a ’theory of change’. These models recognise that evidence is only one part of policy influence and that positive influence of policy is highly dependent on context.

A rather low scientific productivity in terms of publications has been seen after the Ecohealth projects finished. However, it is common for 3–4-year-long research projects to produce most publications 5–8 years after project completion so it is too early to evaluate this aspect. There is some evidence that the network and capacity developed from Ecohealth projects helped acquire new projects, as is the case of phase 2 of the APEIR. The latter now has two new projects on wildlife and antimicrobial resistance that are still fully funded by the IDRC. The translational aspect of these projects looks limited except for the steering committee of the APEIR composed by senior members in member countries. Nevertheless, Ecohealth is not at the level where it is accepted widely as a valid approach to research and intervention.

Finally, capacity building in Ecohealth at different levels, namely from individual to institutional levels, is needed in the region and sensitising policy makers on Ecohealth so that this approach could be happen at a larger scale in research, teaching and intervention. It is known that there is lots of thinking and discussion on Ecohealth and One Health, however, more concrete case studies using Ecohealth and showing added value of this approach are urgently needed. This is also the way to bridge the gap between research and practice in Ecohealth.

## Endnote

^a^Outcome mapping (OM) is a methodology for planning and assessing development programming that is oriented towards change and social transformation. OM provides a set of tools to design and gather information on the outcomes, defined as behavioural changes, of the change process (http://www.outcomemapping.ca).

## Electronic supplementary material

Additional file 1: **Multilingual abstracts in the six official working languages of the United Nations.** (PDF 236 KB)
