# Supplementary material for: Ecohealth research in Southeast Asia: past, present and the way forward
Source: Infect Dis Poverty. 2015 Jan 29;4:5. doi: 10.1186/2049-9957-4-5 (PMC4429815; doi:10.1186/2049-9957-4-5)
Supplement: Supplementary file 1 — Additional file 1: Multilingual abstracts in the six official working languages of the United Nations. (PDF 236 KB) [file 40249_2014_97_MOESM1_ESM.pdf]

Translation of the abstract into the six official working languages of the United Nations

أبحاث الصحة البيئية "Ecohealth" في منطقة جنوب شرق آسيا: الماضي والحاضر والسبيل للمستقبل

هونغ نجوين-نام، سيوبهان دوريا، دينه شوان تونغ، هاين مالي، بروس ويلكوكس، ديليا جريس

ملخص

الصحة البيئية "Ecohealth" هي منهج شامل لفهم الصحة من جوانبها الإنسانية والحيوانية والبيئية في سياق الأنظمة الاجتماعية البيئية. وقد انتشر هذا المنهج بصورة واسعة في جنوب شرق آسيا، حيث قام المركز الدولي الكندي لأبحاث التنمية (IDRC) بإطلاقه في أواخر الألفية الثانية. وبهدف التخلص من الأمراض المعدية الناشئة، تم إطلاق العديد من المشاريع والأنشطة على مستوى المنطقة. وتتشابه الصحة البيئية "Ecohealth" بشكل كبير مع منهج صحة واحدة "One Health"، حيث يشدد المنهجان على أهمية الفهم الشامل للصحة.
وقد قمنا بإجراء دراسة نطاق شملت كافة برامج الصحة البيئية "Ecohealth" والمبادرات والمشاريع التي تم تنفيذها في منطقة جنوب شرق آسيا منذ إطلاق المنهج، كما تم جمع معلومات من أبحاث نظراء مختصين. ويكمن الهدف من هذه الورقة البحثية في استعراض أنشطة الصحة البيئية "Ecohealth" في منطقة جنوب شرق آسيا خلال العشر سنوات الماضية لتسليط الضوء على الدروس المستفادة والتحديات وسبل تقدم الصحة البيئية "Ecohealth" في المنطقة مستقبلاً.
وقد شملت الأنشطة التي تم تنفيذها أنشطة تركز بشكل خاص على الموارد، ومشاريع تركز على الأبحاث، وتلك التي تركز على الجانبين. وتضم الإنجازات إلى الآن بحثاً حول الأمراض المعدية في سياق العوامل الاجتماعية البيئية وجوانب التكثيف الحضري والزراعي ذات الصلة.
إلا أنه ما زال هناك تحد في تصميم المشروع ومستوى التنفيذ في ضوء الموارد المتاحة، والتنسيق لتوظيف فريق عمل لأبحاث الصحة البيئية "Ecohealth" في الدول المعنية، وقياس مدى استيعاب فرق العمل لمبادئ الصحة البيئية "Ecohealth" الأساسية وكيفية ترجمتها للسيطرة على الأمراض وتحقيق وقاية مستدامة منها، ذلك بالإضافة إلى القدرة على زيادة مشاريع الصحة البيئية "Ecohealth".
ونرى بأن السبل المستقبلية للصحة البيئية "Ecohealth" لابد أن تعتمد على منظور إقليمي فيما يتعلق بالأبحاث والتدريب وترجمة السياسات بالاعتماد على منهجي الصحة البيئية "Ecohealth" وصحة واحدة "One Health".

Translated from English version into Arabic by noor86i, through

TRANSLATORS
WITHOUT BORDERS

东南亚生态健康研究的过去、现在和将来

Hung Nguyen-Viet, Siobhan Doria, Dinh Xuan Tung, Hein Mallee, Bruce A Wilcox, Delia Grace

摘要

生态健康是通过分析社会生态系统中人、动物和环境之间的关系，对健康做出全面理解的一种综合性方法。2000 年末，为了解决新发传染病（EIDs）的难题，加拿大国际发展研究中心(IDRC)在东南亚（SEA）地区开展了大量的课题研究，生态健康方法在东南亚地区得到了广泛地推广和应用。生态健康方法与世界卫生组织提出的同一个世界同一个健康的观念（One World One Health）日益融合，两者都强调对健康的整体理解。我们对在东南亚地区开展的所有生态健康研究项目以及相关的同行评议的文献资料进行一个综述，目的是通过回顾近 10 年来在东南亚地区开展的所有生态健康研究项目及活动，总结我们所取得的经验、面对的挑战以及未来的发展方向。生态健康研究从一开始主要关注于能力培养到后来逐步关注项目重点研究及项目覆盖范围。迄今取得的成就主要包括社会生态因素、相关的城市化和农业集约化与传染病领域的相互

关系。主要面临的挑战主要是在项目设计和实施层面，包括发展各个国家生态健康研究团队的能力和协作精神、对团队生态健康基本原则的同化性的评价、研究成果如何转换为可持续的疾病预防和控制措施以及逐步增加生态健康研究项目的能力等。未来推进生态健康的方法应当是从不同研究区域的角度，以生态健康和一个健康（One Health）相融合的观念，进行培训和政策传播。

Translated from English version into Chinese by Tian Li-guang, through

CHINESE CENTER FOR DISEASE CONTROL AND PREVENTION
NATIONAL INSTITUTE OF PARASITIC DISEASES

Recherche Eco-santé en Asie du Sud-Est: passée, présente et à venir

Hung Nguyen-Viet, Siobhan Doria, Dinh Xuan Tung, Hein Mallee, Bruce A Wilcox, Delia Grace

Résumé
Eco-santé est une approche complète pour comprendre la santé dans son interface humaine, animale et environnementale dans un contexte de systèmes socio-écologiques. Cette approche a été largement introduite en Asie du Sud-Est par le Centre de Recherche de Développement International Canadien (CRDI) à la fin des années 2000. Destinés à aborder le problème des maladies infectieuses émergentes (MIE), plusieurs de ces projets et activités ont été conçus dans toute la région. Eco-santé converge de plus en plus sur l'approche d' "une seule santé", car les deux mouvements mettent l'accent sur une compréhension holistique de la santé. Nous avons réalisé un examen exploratoire en prenant en considération tous les programmes Eco-santé, les initiatives et les projets mis en œuvre en Asie du Sud-Est depuis l'introduction de l'approche, et nous avons également rassemblé des informations recueillies dans des publications approuvées par des collègues. L'objectif de cet article est d'examiner les activités Eco-santé en Asie du Sud-Est au cours des dix dernières années, afin de faire bon usage des leçons apprises, des obstacles rencontrés et de la marche en avant d'Eco-santé dans la région. Les activités vont de celles purement axées sur la capacité, aux projets focalisés sur la recherche et aux projets couvrant les deux. Les réalisations accomplies jusqu'à présent incluent, par exemple, la contribution à la recherche dans le domaine des maladies infectieuses en relation avec des facteurs écologiques sociaux, et à l'urbanisation et l'intensification de l'agriculture associées. Des obstacles demeurent au niveau de la création du projet et de la mise en œuvre, dans la capacité et la coordination disponibles pour développer des équipes de recherche Eco-santé dans les pays, l'assimilation d'équipes de gestion des principes de base d'Eco-santé et leur traduction en une prévention et un contrôle durable des maladies, ainsi que la possibilité de développer les projets Eco-santé. Nous suggérons que la marche en avant d'Eco-santé se fasse suivant une perspective régionale en termes de recherche, de formation et de traduction d'une politique utilisant Eco-santé en association avec l'approche "Sante unique».

Translated from English version into French by Ode Laforge, through

TRANSLATORS
WITHOUT BORDERS

Исследование концепции «Экология и здоровье» в Юго-Восточной Азии: прошлое, настоящее и пути дальнейшего развития

Ханг Нгуен-Вьет, Сёбхан Дория, Дин Ксуан Тунг, Хейн Мали, Брюс А. Уилкокс, Делия Грейс

Аннотация

Концепция «Экология и здоровье» (Ecohealth) представляет собой комплексный подход к пониманию здоровья со стороны человека, животных и окружающей среды в контексте социально-экологических систем. Данный подход был широко внедрен в Юго-Восточной Азии (ЮВА), Канадским международным научно-исследовательским центром развития (IDRC) в конце 2000-х гг. По всему региону были созданы подобные многочисленные проекты и мероприятия, направленные на решение проблем новых инфекционных заболеваний (EIDs). Концепция «Экология и здоровье» все больше и больше сближается с концепцией «Неделимое здоровье», так как оба движения подчеркивают целостное понимание здоровья. Мы провели обзор, рассматривая все программы, инициативы и проекты концепции «Экология и здоровье», которые были осуществлены в ЮВА после внедрения концепции, а также собрали информацию из рецензируемой литературы. Целью настоящей статьи является обзор мероприятий концепции «Экология и здоровье» в ЮВА за последние 10 лет для обращения к полученным урокам, проблемам, с которыми столкнулась концепция, и путей дальнейшего развития в регионе. Мероприятия варьируются от проектов, фокусирующихся только на потенциале, до проектов, фокусирующихся на исследованиях и проектов, охватывающих и то и другое. На сегодняшний день, достижения включают, например, научно-исследовательский вклад в области инфекционных заболеваний с учетом социально-экологических факторов и связанной урбанизацией, и интенсификацией сельского хозяйства. Проблемы остаются на уровне разработки и реализации проекта при имеющихся возможностях и координации для развития в странах исследовательских групп концепции «Экология и здоровье» с оценкой освоения группами лежащих в основе принципов концепции и их перевод в устойчивые методы профилактики и контроля заболеваний, а также возможность масштабирования проектов концепции «Экология и здоровье». Мы считаем, что пути дальнейшего развития концепции «Экология и здоровье» должны осуществляться с учетом региональной перспективы относительно научных исследований, профессиональной подготовки и перевода политики, используя концепцию «Экология и здоровье» в сочетании с концепцией «Неделимое здоровье».

Translated from English version into Russian by Alexander Vareiko, through

TRANSLATORS
WITHOUT BORDERS

Investigación ecoepidemiológica en el Sudeste Asiático: pasado, presente y camino correcto

Hung Nguyen-Viet, Siobhan Doria, Dinh Xuan Tung, Hein Mallee, Bruce A Wilcox, Delia Grace

Resumen

La ecoepidemiología ambiental propone entender la salud desde un punto de vista integral en su interfaz humana, animal y medioambiental en el contexto de un sistema socioecológico. El Centro Internacional de Investigación para el Desarrollo (CIID) de Canadá introdujo este enfoque de forma general en el Sudeste Asiático a finales de la década de 2000. Con el fin de abordar el problema de las enfermedades infecciosas emergentes (EID), se han generado numerosos proyectos y actividades similares en toda la región. La ecoepidemiología converge cada vez más con el enfoque Una Sola Salud, ya que ambos movimientos hacen hincapié en una comprensión integral de la sanidad. Hemos realizado un estudio de alcance al tener en cuenta todos los programas, iniciativas y proyectos ecoepidemiológicos que se han ejecutado en el SA desde la introducción de dicho enfoque, además de recopilar información de literatura revisada por pares. El objetivo del presente artículo es analizar las actividades ecoepidemiológicas en el SA en el transcurso de los últimos 10 años para tratar las lecciones aprendidas, los obstáculos afrontados y el camino correcto a seguir en ecoepidemiología en la región. Las actividades varían desde aquellas orientadas exclusivamente a las capacidades, proyectos centrados en la investigación y los que cubren ambos. Entre los logros conseguidos hasta la fecha se incluye, por ejemplo, la investigación que contribuye al campo de las enfermedades infecciosas en relación con factores socioecológicos y la urbanización e intensificación que conllevan. Aún quedan dificultades a nivel de diseño y ejecución del proyecto, en la capacidad disponible y en la coordinación en el desarrollo de equipos de investigación en ecoepidemiología en los países, en la estimación de la integración por parte de los equipos de los principios subyacentes de la ecoepidemiología y su aplicación a una prevención y control de enfermedades sostenible, así como en la capacidad de perfeccionar los proyectos ecoepidemiológicos. Sugerimos una perspectiva regional como camino a seguir en ecoepidemiología en cuanto a investigación, formación y aplicación de políticas, utilizando la ecoepidemiología en combinación con el enfoque Una Sola Salud.

Translated from English version into Spanish by Raquel Bentué, through

TRANSLATORS
WITHOUT BORDERS
